# GBMPurity: A machine learning tool for estimating glioblastoma tumor purity from bulk RNA-sequencing data

**DOI:** 10.1093/neuonc/noaf026

**Published:** 2025-02-01

**Authors:** Morgan P H Thomas, Shoaib Ajaib, Georgette Tanner, Andrew J Bulpitt, Lucy F Stead

**Affiliations:** Leeds Institute of Medical Research at St James’s, University of Leeds, Leeds, UK; School of Computer Science, University of Leeds, Leeds, UK; Leeds Institute of Medical Research at St James’s, University of Leeds, Leeds, UK; Leeds Institute of Medical Research at St James’s, University of Leeds, Leeds, UK; School of Computer Science, University of Leeds, Leeds, UK; Leeds Institute of Medical Research at St James’s, University of Leeds, Leeds, UK

**Keywords:** deconvolution, glioblastoma, transcriptomics, tumor microenvironment, tumor purity

## Abstract

**Background:**

Glioblastoma (GBM) presents a significant clinical challenge due to its aggressive nature and extensive heterogeneity. Tumor purity, the proportion of malignant cells within a tumor, is an important covariate for understanding the disease, having direct clinical relevance or obscuring signal of the malignant portion in molecular analyses of bulk samples. However, current methods for estimating tumor purity are nonspecific and technically demanding. Therefore, we aimed to build a reliable and accessible purity estimator for GBM.

**Methods:**

We developed GBMPurity, a deep learning model specifically designed to estimate the purity of IDH-wild type primary GBM from bulk RNA-sequencing (RNA-seq) data. The model was trained using simulated pseudobulk tumors of known purity from labeled single-cell data acquired from the GBmap resource. The performance of GBMPurity was evaluated and compared to several existing tools using independent datasets.

**Results:**

GBMPurity outperformed existing tools, achieving a mean absolute error of 0.15 and a concordance correlation coefficient of 0.88 on validation datasets. We demonstrate the utility of GBMPurity through inference on bulk RNA-seq samples and observe reduced purity of the proneural molecular subtype relative to the classical, attributed to the increased presence of healthy brain cells.

**Conclusions:**

GBMPurity provides a reliable and accessible tool for estimating tumor purity from bulk RNA-seq data, enhancing the interpretation of bulk RNA-seq data and offering valuable insights into GBM biology. To facilitate the use of this model by the wider research community, GBMPurity is available as a web-based tool at: https://gbmdeconvoluter.leeds.ac.uk/.

## Graphical Abstract

**Graphical Abstract:**
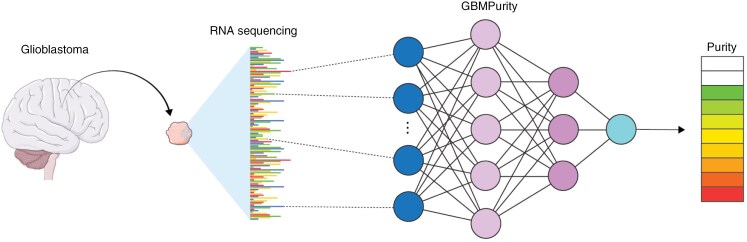

**Key Points**

- GBMPurity is a glioblastoma-specific purity estimation tool.
- The model accurately estimates the purity of bulk RNA-sequencing data, outperforming existing tools.
- The model is available online at: https://gbmdeconvoluter.leeds.ac.uk/.

**Importance of the Study**

Glioblastoma (GBM) is a deadly brain tumor with a dismal prognosis. Research on this disease has lagged compared to other cancers, underscoring the need to streamline investigations. The cellular composition of the GBM tumor microenvironment significantly influences therapy resistance, prognosis, and the molecular state of neoplastic cells. Consequently, tumor purity (the proportion of malignant cells within a tumor) is a critical variable for understanding and contextualizing molecular and clinical analyses. We present GBMPurity (https://gbmdeconvoluter.leeds.ac.uk/), an accessible, GBM-specific tool that accurately predicts sample purity from bulk RNA-sequencing data. This tool can be used by the wider research community to support the interpretation of bulk omics data and accelerate the identification of more effective therapeutic strategies for treating GBM.

Glioblastoma (GBM) is the most common and aggressive primary brain tumor in adults, with a median survival time between 10 and 14 months and only 30% of patients surviving beyond 1 year.^1^ This dismal prognosis is due to GBM’s rapid growth and diffuse nature combined with a lack of therapeutic innovation, with no significant advancements in treatment strategies for 2 decades.^2^

The complexity of GBM, characterized by substantial inter- and intra-tumoral heterogeneity in genetic, epigenetic, and cellular landscapes, poses significant challenges to understanding the disease and identifying consistent therapeutic targets.^3–6^ Moreover, brain tumor research has been historically underfunded, resulting in slower progress compared to other cancers.^7^ This underscores the urgent need to streamline GBM research to better understand its biology and develop more effective treatments.

The GBM tumor microenvironment (TME) is highly heterogeneous and has a well-described effect on malignant progression. It includes a variety of nonmalignant cells such as neurons, astrocytes, oligodendrocytes, microglia, infiltrating immune cells, and vasculature. These components influence the state and evolution of GBM cells and contribute to the malignant phenotype, radiotherapy resistance, and overall prognosis.^8–12^

Bulk omics data provides a composite view of all cells within a sample, making tumor purity—a measure of the ratio of malignant to nonmalignant cells—a critical factor in data interpretation. Low tumor purity can obscure meaningful signals from the malignant cell fraction, complicating genomic analysis and masking clinical insights.^13,14^ Single-cell approaches can overcome this issue, but they are technically challenging and costly, limiting their application to sufficient tissue sample numbers for ascertaining biological and clinical insights in this heterogeneous disease. Consequently, accurately quantifying the contribution of malignant cells to bulk omics data serves as a crucial covariate for deciphering malignant-cell-intrinsic biology.

The purity of a bulk tumor sample can be quantified prior to any molecular analysis by pathology assessment, but these estimates can vary significantly.^15^ Genomic-based methods, which compare somatic CNA between malignant and nonmalignant cell components, offer an alternative. However, these methods do not facilitate purity inference on publicly available or newly generated bulk RNA-sequencing (RNA-seq) datasets where matched DNA sequencing is not available. Therefore, RNA-based purity prediction methods are needed.

Purity estimation can be framed as a cellular deconvolution problem with 2 cell types, malignant and nonmalignant. While there exist multiple RNA-seq compositional deconvolution tools such as CIBERSORTx,^16^ MuSiC,^17^ and Scaden,^18^ applying these deconvolution tools can be time-consuming and challenging, particularly for bioinformatics-naïve investigators. Revkov et al^19^ have developed a pan-cancer purity estimation tool, PUREE, based on consensus genomic-derived purity labels. However, since different tissues and cancers contain distinct cell types and therefore specific expression profiles,^20^ we posit that tissue-specific tools will demonstrate improved accuracy.

To test this, we aimed to build and optimize a tissue-specific purity estimator for GBM, a cancer where purity is particularly pertinent for understanding molecular disease states.^6^ Our approach leverages deep learning combined with ad hoc simulation of pseudobulk tumors of known purity from single-cell data.

We demonstrate that our approach outperformed general tools: PUREE, CIBERSORTx, MuSiC, and Scaden. Therefore, we developed the model into a “plug-and-play” web-based tool called GBMPurity, which is freely available at https://gbmdeconvoluter.leeds.ac.uk/. Users simply upload raw count data of bulk RNA-seq GBM samples and receive accurate estimations of sample purity.

## Methods

### Data Acquisition and Preprocessing

#### Bulk.—

Raw, longitudinally matched IDH-wild type primary GBM tissue samples were acquired from various sources, with bulk RNA-seq performed according to the protocol described by Tanner et al. We acquired additional raw RNA-seq data from several published studies, following Data Transfer Agreements where required.^21–26^ The resulting FASTQ files were processed into a count matrix using the pipeline detailed by Tanner et al. Additionally, we obtained preprocessed RNA-seq data, in the form of transcript counts, from the GLASS consortium via their portal (https://www.synapse.org/glass).^26^ This formed our “Discovery” cohort of 258 bulk samples. We also obtained raw BAM files and matching DNA-based purity estimates for 260 tumors from the EORTC cohort,^27^ which were processed via the same pipeline as the Discovery cohort. For this EORTC cohort, RNA, and DNA were extracted via combined isolations from FFPE samples using the AllPrep DNA/RNA FFPE kit (Qiagen).^27^ A further 144 tumors from the TCGA were downloaded via the TCGAbilonks R package (v 2.32.0)^28,29^ with ABSOLUTE purity estimates for these tumors obtained from Ceccarelli et al.^30^ Finally, another 109 samples from the CGGA were downloaded from http://www.cgga.org.cn/download.jsp.^31^ Details of these datasets can be found in Table 1.

**Table 1.** Glioblastoma Datasets Used in This Article

Data type	Dataset	Accession(s)	Samples (used in this study)	Platform	Reference
Bulk	Stead	EGAD00001009806 (https://ega-archive.org/datasets/EGAD00001009806)	64 bulk tumor samples from 32 matched primary and recurrent pairs	Illumina NGS paired-end stranded total RNA	Tanner et al^6^
	Additional stead	Unpublished and available from the corresponding author via data access agreement	42 bulk tumor samples from 21 matched primary and recurrent pairs	Illumina NGS paired-end stranded total RNA	Unpublished
	GLASS	The GLASS Consortium (https://www.synapse.org/#!Synapse:syn31121291)	46 bulk tumor samples from 23 matched primary and recurrent pairs	Illumina HiSeq 4000 stranded paired-end mRNA	Varn et al^26^
	Nam	EGAD00001001424 (https://ega-archive.org/datasets/EGAD00001001424)	70 bulk tumor samples from 35 matched primary and recurrent pairs	Illumina HiSeq 2000 unstranded paired-end mRNA	Kim et al^21^
	Kim	PRJNA580196 (https://www.ncbi.nlm.nih.gov/bioproject/?term=PRJNA580196)	26 bulk tumor samples from 13 matched primary and recurrent pairs	Illumina NextSeq 500 paired-end stranded total RNA	Kim et al^24^
	DFKZ	EGAD00001004564 (https://ega-archive.org/datasets/EGAD00001004564)	32 bulk tumor samples from 16 matched primary and recurrent pairs	Illumina HiSeq 2000 paired-end stranded total RNA	Körber et al^23^
	Rabadan	EGAD00001002143 (https://ega-archive.org/datasets/EGAD00001002143)	16 bulk tumor samples from 8 matched primary and recurrent pairs	Illumina TruSeq stranded paired-end total (14), mRNA (2)	Wang et al^22^
	Diaz	EGAS00001004524 (https://ega-archive.org/studies/EGAS00001004524)	8 bulk tumor samples from 4 matched primary and recurrent pairs	Illumina NovaSeq paired-end unstranded mRNA	Wang et al^25^
	EORTC	EGAD00001007860 (https://ega-archive.org/datasets/EGAD00001007860)	260 bulk tumor samples from 130 matched primary and recurrent pairs	Illumina NovaSeq stranded paired-end total RNA	Hoogstrate et al^27^
	TCGA	TCGAbiolinks R package^28^	144 bulk tumor samples	Illumina HiSeq 4000 stranded paired-end mRNA	Ceccarelli et al^29^
	CGGA	http://www.cgga.org.cn/download.jsp	109 bulk tumor samples	Illumina HiSeq 2000, 2500, or 4000 paired-end total RNA	Zhao et al^31^
Single-cell	Wang	GSE174554 (https://www.ncbi.nlm.nih.gov/geo/query/acc.cgi?acc=GSE174554)	245 952 single-nuclei from 57 samples including 22 matched primary and recurrent pairs	10X Genomics, Illumina NovaSeq 6000	Wang et al^11^
	Neftel	GSE131928 (https://www.ncbi.nlm.nih.gov/geo/query/acc.cgi?acc=GSE131928)	16 201 single-cells from 9 patients	10X Genomics Chromium 3’ single-cell	Neftel et al^5^
	GBmap	https://cellxgene.cziscience.com/collections/999f2a15-3d7e-440b-96ae-2c806799c08c	890892 single-cells and 244 785 single-nuclei from 240 patients across 26 studies	Various	Ruiz-Moreno et al^32^

#### Single-cell RNA-seq data.—

The training single-cell RNA-seq (scRNA-seq) dataset was obtained from the extended GBmap resource (https://cellxgene.cziscience.com/collections/999f2a15-3d7e-440b-96ae-2c806799c08c), an integrated and annotated single-cell atlas of IDH-wild type primary GBM encompassing over 1.1 million cells from 240 patients across 26 studies.^32^ The data collated in GBmap comprise a mixture of both single-cell and single-nuclei (snRNA-seq) experiments. These data were prefiltered by the original authors for cells that expressed over 500 genes, 1000 RNA counts, and less than 30% mitochondrial reads.

Two validation IDH-wild type primary GBM datasets were utilized. The first snRNA-seq dataset from Wang et al. was downloaded from GSE174554^11^ (*n* = 57) and processed using Seurat’s (v5.1.0) SCTransform method. Malignant cells in this dataset were pre-annotated by the original authors.

The second scRNA-seq dataset from Neftel et al was retrieved from GSE131928.^5^ This dataset comprises a mixture of Smart-seq2 and 10× single-cell profiling data. Given that the Smart-seq2 data were enriched for malignant cells, only the 10× data were included in this study to ensure a range of purities for model evaluation (*n* = 9). As this dataset is part of GBmap, samples used for validation were excluded from the GBmap dataset to avoid data leakage. This data was filtered using Seurat (v5.1.0) for cells with more than 800 RNA counts, over 200 detected genes, and less than 5% mitochondrial gene content. Doublets were identified and removed using the DoubletFinder package (v2.0.4)^33^ (Supplementary Figure S1A). The single-cell data were then integrated using Seurat’s (v5.1.0) IntegrateLayers with the CCAIntegration method after applying the NormalizeData, FindVariableFeatures, and RunPCA methods with default parameters (Supplementary Figure S1B, C).

#### Cell-type assignment.—

Malignant cells were identified through copy number alteration (CNA) analysis using the CONICSmat package (v0.0.0.1).^34^ Cells were annotated as malignant if they had a posterior probability greater than 0.95 of harboring one of the GBM chromosomal aberrations: chromosome 7 arm p and q amplification or chromosome 10 arm q deletion (Supplementary Figure S1D).

Cells labeled as normal following CNA annotation were then assigned a nonmalignant cell label using the scanpy (v1.9.8)^35^ ingest method after applying the normalize_total, log1p, and scale preprocessing methods. The GBmap nonmalignant cells derived from single-nuclei experiments were used as the reference.

#### Pseudobulking.—

Pseudobulking was performed by summing the gene expression profile of all cells belonging to a sample:


$$\begin{array}{l} Pseudobulk_{js}=\overset{Cs}{\underset{i=1}{\sum }}x_{ij}\;\;\; \end{array}$$

where $Pseudobulk_{js}$ refers to the pseudobulk of gene *j* in sample *s*, $x_{ij}$ is the raw count of the gene j of the ith cell, and Cs are all cells in sample s. The ground truth purity of these pseudobulks is then the fraction of malignant cells assigned to that pseudobulk.

#### Molecular subtyping.—

For the molecular subtyping of bulk and pseudobulk tumors, a matrix of transcript per million (TPM) normalized protein-coding genes was uploaded to the GlioVis web application (http://gliovis.bioinfo.cnio.es/).^36^ The tumors were then classified according to the consensus of the 3-way SubtypeME tool.

The TPM normalization was performed using the following equation:


$$\begin{array}{l} TPM_{ij}=\frac{\frac{x_{ij}}{l_{i}}}{\overset{n}{\underset{k=1}{\sum }}\frac{x_{kj}}{l_{k}}}\times 10^{6} \end{array}$$

where $x_{ij}$ is the raw count of the ith gene in the jth sample, and $l_{i}$ is the length of the ith gene.

#### Cell type deconvolution.—

For the deconvolution of bulk samples, the TPM normalized protein-coding genes were uploaded to the GBMDeconvoluteR web application (https://gbmdeconvoluter.leeds.ac.uk/).^37^ The deconvolution was performed using the Ruiz-Moreno marker gene list.

### GBMPurity Model Development

#### Feature selection.—

To develop the GBMPurity model aimed at inferring the purity of bulk RNA-seq GBM samples using scRNA-seq data, our feature selection process focused on identifying genes that are consistently represented across both RNA-seq modalities. We compared bulk RNA-seq data to the pseudobulked GBmap single-cell data. We first excluded genes that were either absent or expressed at low levels (counts per million [CPM] < 1 in 50% of samples) in either modality.

The CPM normalization was performed using the following equation:


$$\begin{array}{l} CPM_{ij}=\frac{x_{ij}}{\overset{n}{\underset{k=1}{\sum }}x_{kj}}\times 10^{6} \end{array}$$

where $x_{ij}$ is the raw count of the ith gene in the jth sample.

Following CPM normalization, we employed the Kolmogorov–Smirnov (KS) statistic to quantify the distance between the empirical distribution functions of each gene in the 2 modalities, providing a measure of similarity between the distributions. Genes with a KS statistic above an arbitrary threshold of 0.4 were excluded, resulting in a final set of 5829 genes for model training (Supplementary Figure S2).

#### Simulation of pseudobulk samples.—

Deep learning models typically contain a large number of learnable parameters, enabling them to capture highly complex functions. However, this also makes them prone to overfitting in the absence of extensive training data. To mitigate this risk, we simulated additional training samples using the single-cell data by random sampling of cells over a range of malignant to nonmalignant ratios before pseudobulking. This was performed ad hoc during the training process, which allowed us to generate sufficient training data.

We preserve interpatient heterogeneity by sampling cells only from within the same GBM sample. Samples containing fewer than 5 malignant or nonmalignant cells were excluded to ensure an adequate number of cells for sampling across the purity spectrum.

To simulate pseudobulk tumors for a given sample i, 2 random numbers are generated: the target purity p (0–1), and the number of cells N (200–4000). Based on p and N, the number of malignant ($N_{m}$) and nonmalignant ($N_{n}$) cells are calculated. Cells (x) are randomly sampled with replacements from the selected sample. The RNA-seq counts are summed across the sampled malignant and nonmalignant cells to simulate a pseudobulk tumor:


$$\begin{array}{l} Pseudobulk\;\;\;Counts=\overset{N_{m}}{\underset{k=1}{\sum }}x_{m,k}^{\left(i\right)}+\overset{N_{n}}{\underset{l=1}{\sum }}x_{n,l}^{\left(i\right)} \end{array}$$

#### Input data processing.—

Simulated pseudobulk samples undergo several transformation steps to prepare the data for model training. The steps are as follows:

1. The raw counts of the simulated pseudobulk samples are first TPM transformed.
2. The TPM values are then divided by 100 to rescale the data to a more suitable range for model training.
3. A log_2_ transformation is applied to the scaled TPM values after adding 1 pseudocount to each value to avoid infinite values:

$$\begin{array}{l} \log_{2}\left(\frac{TPM_{ij}}{100}+1\right) \end{array}$$

#### Model construction.—

GBMPurity is a regression machine learning model developed using PyTorch (v2.2.0), designed to predict the purity of bulk GBM samples from an input of 5829 selected genes. The model outputs a single numeric value representing the estimated purity. It was trained using the Adam optimizer, processing data in batches of 64 randomly simulated pseudobulks until convergence of the L1 loss function. L1 loss was chosen to reduce the impact of outliers during model training, as it helps stabilize weight updates in the presence of noise and variability in malignant-cell assignments for ground truth purity labels. Convergence was defined as the point where the average training loss failed to decrease over a sliding window of 25 batches.

L1 loss is defined as:


$$\begin{array}{l} L1\left(y,\hat{y}\right)=\frac{1}{n}\overset{n}{\underset{i=1}{\sum }}\left|y_{i}-\hat{y_{i}}\right| \end{array}$$

where $y_{i}$ is the actual purity of ith sample and $\hat{y_{i}}$ is the predicted purity.

The model’s performance relies on several hyperparameters which were optimized through cross-validation. Each dataset in the GBmap resource was treated as an individual fold. For effective evaluation across a representative spectrum of purity, we focused on 11 of the 26 folds that contained at least 5 samples with purity values between 0.1 and 0.9. These 11 folds were used as holdout datasets, resulting in 11 cross-validation iterations.

The following hyperparameters were tuned sequentially in independent experiments (as shown in Supplementary Figure S3): the number of hidden layers, the size of the hidden layer dimensions, the dropout rate, weight decay, learning rate, and patience (defined as the number of batches to wait for a decrease in loss before terminating training). These hyperparameters were fine-tuned to achieve optimal model performance, ensuring robustness and generalizability in predicting the purity of bulk GBM samples.

#### GBMPurity model.—

The resulting GBMPurity model is a multilayer perceptron with 2 hidden linear layers, comprising 32 and 16 neurons respectively, each employing a rectified linear unit (ReLU) activation function. The model was trained with a learning rate of 3e^−5^, a weight decay of 1e^−5^, and an input layer dropout probability of 0.4. We saved the model with the lowest average loss over a 25-batch sliding window and terminated training when this sliding average did not decrease for 200 batches.

During inference, the 5829 selected genes are input into the model. The model outputs a continuous prediction of purity, which can theoretically range from negative infinity to infinity due to the absence of an activation function on the output layer. To ensure meaningful predictions, we manually clip these outputs to a range between 0 and 1.

#### Model evaluation.—

For the evaluation of GBMPurity, along with other benchmarked models described below, we input pseudobulks of true samples from Wang et al and Neftel et al and measure the error of the predictions versus the labeled true purity. We describe the performance across 4 metrics: mean absolute error (MAE), root mean squared error (RMSE), Pearson correlation, and Correlation Concordance Correlation (CCC):


$$\begin{array}{l} MAE=\frac{1}{n}\overset{n}{\underset{i=1}{\sum }}\left|y_{i}-\hat{y_{i}}\right| \end{array}$$

$$\begin{array}{l} RMSE=\sqrt{\frac{1}{n}\overset{n}{\underset{i=1}{\sum }}{\left(y_{i}-\hat{y_{i}}\right)}^{2}} \end{array}$$

$$\begin{array}{l} Pearson=\frac{\overset{n}{\underset{i=1}{\sum }}\left(y_{i}-\bar{y}\right)\left(\hat{y_{i}}-\bar{\hat{y}}\right)}{\sqrt{\overset{n}{\underset{i=1}{\sum }}{\left(y_{i}-\bar{y}\right)}^{2}}\;\sqrt{\overset{n}{\underset{i=1}{\sum }}{\left(\hat{y_{i}}-\bar{\hat{y}}\right)}^{2}}} \end{array}$$

$$\begin{array}{l} CCC=\frac{2\sigma_{y\hat{y}}}{\sigma_{y}^{2}+\sigma_{\hat{y}}^{2}+{\left(\bar{y}-\bar{\hat{y}}\right)}^{2}} \end{array}$$

where $y_{i}$ is the actual value and $\hat{y_{i}}$ is the predicted value of the ith sample, and n is the number of observations.

### Model Interpretation

To understand and validate the predictions of GBMPurity, we employed techniques for feature attribution and model interpretation.

#### SHapley Additive exPlanations*.—*

We utilized SHapley Additive exPlanations (SHAP) to determine each feature’s impact on the predicted purity in the pseudobulked training data.^38^ This was implemented using the DeepExplainer class from the Python shap package (v0.45.1). SHAP values provide a measure of each gene’s contribution to the model’s output, allowing for a detailed understanding of feature importance.

#### Gene set enrichment analysis.—

Following SHAP, we generated a pre-ranked list by averaging the SHAP contributions for each feature across the training data. Gene set enrichment analysis (GSEA) was then performed using the brain-related gene set database curated by Hagenauer et al^39^ with the fgsea package (v1.26.0) in R.^40^ This analysis was conducted without ranking metric weighting.

#### Interpretation of hidden nodes.—

To further interpret the internal workings of the model, we used the LayerConductance class from the Python package Captum (v0.7.0) to quantify the average contribution of each hidden neuron to the model output.^41^

### Benchmarking

We compared the performance of GBMPurity against 4 established tools that enable purity prediction. Where single-cell references were necessary for training the model, we used the GBmap dataset with the same 5829 genes and cells labeled as malignant or nonmalignant, mirroring the training process of GBMPurity. The predicted contribution of the malignant component was taken as the model’s prediction of tumor purity. Each model was evaluated using MAE, RMSE, Pearson correlation, and CCC.

#### PUREE.—

PUREE^19^ is a pan-cancer purity estimation tool that employs a weakly supervised machine learning model trained on RNA-seq data across multiple cancer types labeled with consensus purity estimates derived from 4 different algorithms. This pre-trained model did not require single-cell reference data. For purity estimation, pseudobulks of the 3 single-cell datasets used in this study were TPM transformed and uploaded to the PUREE web interface (https://puree.genome.sg/).

#### CIBERSORTx.—

CIBERSORTx^16^ is a cell deconvolution algorithm that uses single-cell reference data to generate gene expression profiles of various cell types. It employs support vector regression to estimate the proportions of different cell types in an RNA-seq mixture. Due to the size of our single-cell reference, CIBERSORTx was run via Docker and used the default parameters.

#### MuSiC.—

MuSiC^17^ deconvolves bulk RNA-seq data using similar means to CIBERSORTx, but instead utilizes sample information to weight genes with consistent cross-subject and cross-cell type consistency, employing a nonnegative least squares algorithm. The deconvolution was performed in R using the music_prop function from the MuSiC package (v1.0.0) with default parameters.

#### Scaden.—

Scaden^18^ is an ensemble deep learning model that deconvolves bulk RNA-seq samples using labeled pseudobulks. This approach is similar to the methods employed in GBMPurity; however, Scaden uses pre-simulated pseudobulks of fixed cell numbers and fixed training steps, whereas GBMPurity generates pseudobulk samples of varying cell numbers during training and terminates training automatically when the loss stops decreasing. We trained Scaden using default parameters on 500 simulated tumors per training sample, each containing 500 cells.

### Statistical Analysis

All statistical analyses were conducted using Python, specifically leveraging the pingouin package (v0.5.4) for statistical computations. Descriptive statistics, inferential tests, and correlation analyses were performed to validate the findings. *P*-values < .05 were considered statistically significant.

### Software and Hardware Use

All computational analyses were performed using ARC4, part of the High-Performance Computing facilities at the University of Leeds, UK. This system runs the CentOS 7 distribution of Linux and contains Intel Xeon Gold 6138 CPUs with up to 768 GB of memory. All analyses were conducted in R version 4.3.1 or Python version 3.10.13.

### Data and Code Availability

The datasets generated and analyzed during the current study are available as described in the original publications. All codes used for data processing, model development, and analysis are available at https://github.com/scmpht/GBMPurity. The pre-trained GBMPurity model, along with instructions for use, is available at https://gbmdeconvoluter.leeds.ac.uk/.

### Ethics Statement

All data used in this study were derived from patients who provided samples with informed, written consent. These samples were approved for use in this study by the UK National Health Service’s Research Ethics Service Committee South Central—Oxford A (Research Ethics Code: 13/SC/0509).

## Results

### Data Collection and Preprocessing

**Figure 1.**
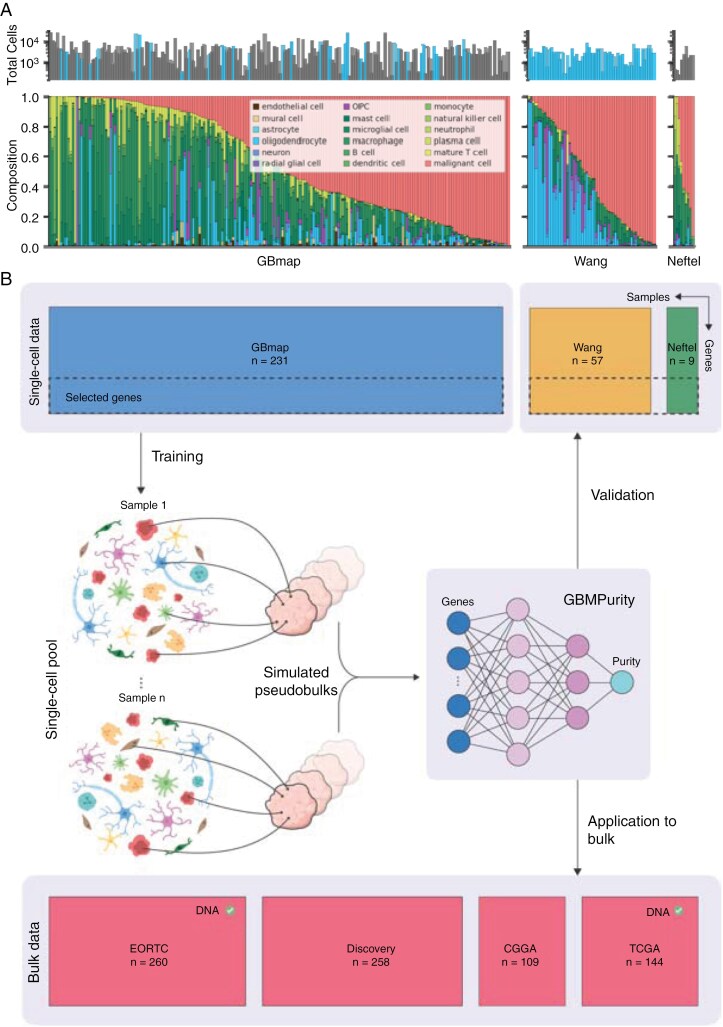
Study design and training methodology for GBMPurity. (A) Sample cell counts (top) and cell type compositions (bottom) in training (GBmap), and validation (Wang and Neftel) datasets are shown. Bars with lighter shade indicate single-nuclei-derived samples, while bars with a darker shade represent single-cell-derived samples. (B) Overview of the GBMPurity training process. Single-cell RNA-seq data from the GBmap dataset (Ruiz-Moreno et al^32^) was compared to bulk GBM RNA-seq data to identify genes with consistent representation across single-cell and bulk modalities (see *Methods* section and Supplementary Figure 2 for details). These selected genes were used to train the GBMPurity model. Within-patient random sampling of cells was performed to simulate pseudobulks with known purity levels, which served as training data for a multilayer perceptron. The trained model was evaluated on 57 single-nuclei pseudobulks from Wang et al^11^ and 9 single-cell pseudobulks from Neftel et al.^5^ Finally, the model was applied to bulk RNA-seq data for purity inference. Datasets marked with a tick had DNA-derived purity estimates available for additional validation of the model’s predictions.

To develop GBMPurity, we emulated bulk tumor expression profiles by pseudobulking labeled single-cell data, a common practice for evaluating deconvolution tools.^42–44^ Since the single-cells are labeled as malignant or nonmalignant we know the ground truth purity of the given pseudobulked tumor. For training, a comprehensive GBM scRNA-seq atlas curated by Ruiz-Moreno et al named GBmap was utilized. This dataset comprises integrated scRNA-seq and snRNA-seq data from 240 GBM patients across 26 studies. Additional validation datasets were obtained from Wang et al, consisting of 57 pre-labeled single-nuclei GBM samples, and Neftel et al, including 9 unlabeled single-cell samples that were manually labeled (Supplementary Figure S1). The Neftel samples were also included in GBmap and thus excluded from the training set, resulting in 231 training and 66 validation samples. Figure 1A displays the included sample cell-type compositions, which due to the use of both single-cell and single-nuclei derived data, contain a range of cell types including those difficult to capture through single-cell experiments such as neurons (Supplementary Table S1).

To ensure the generalizability of our model to bulk RNA-seq samples, we selected genes equally represented across the pseudobulked single-cell GBmap samples and bulk GBM samples. Following CPM normalization, genes with CPM < 1 in over 50% of samples in either modality were excluded. Using a KS statistic threshold of <0.4, we selected 5829 genes with similar distributions across both modalities for model training (Supplementary Figure S2A–D). These selected genes retained multiple canonical markers for each cell type as defined by the OmniPath resource^45^ (Supplementary Figure S2E), and these markers were expressed in the expected cell types in our single-cell training data (Supplementary Figure S2F).

### Model Development

Through random sampling of malignant and nonmalignant cells in our single-cell data, we simulated pseudobulk samples across a range of purities. Simulated pseudobulks were kept within-sample to maintain robustness against intratumoral heterogeneity (Figure 1B). Samples with fewer than 5 malignant or nonmalignant cells were excluded, resulting in 197 samples used for simulation. Pseudobulks were simulated ad hoc during model training until the MAE loss function converged. Hyperparameters were optimized using cross-validation (Supplementary Figure S3), and the final model, which we named GBMPurity, was trained using all 197 training samples. See *Methods* for more information.

### Model Evaluation

**Figure 2.**
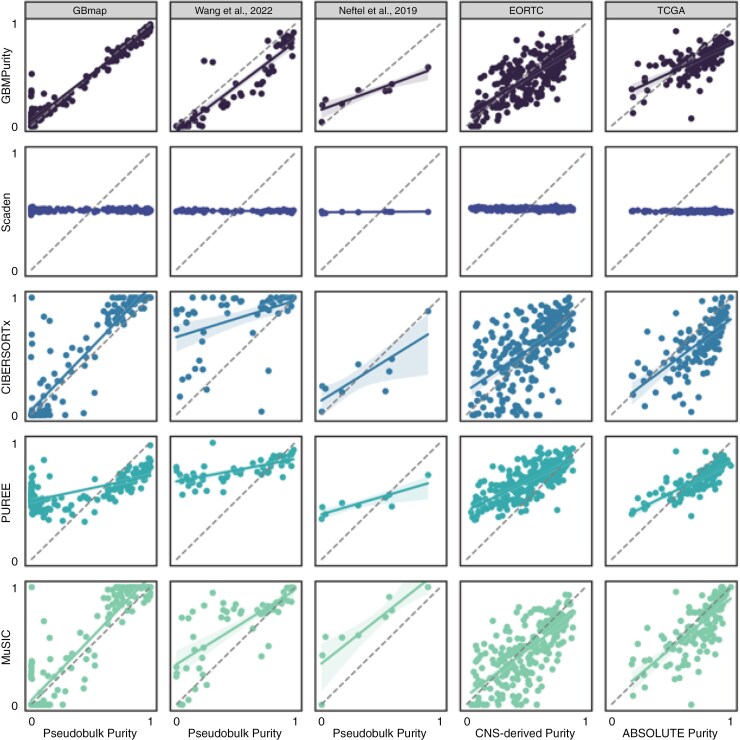
Benchmarking of GBMPurity against alternative tumor purity estimation methods across 5 datasets. The figure displays a 5 × 5 grid summarizing the performance of 5 RNA-based tumor purity estimation methods (rows: GBMPurity, Scaden, CIBERSORTx, PUREE, and MuSiC) across 5 datasets (columns). The first 3 columns represent pseudobulk datasets with ground truth purity labels: GBmap (*n* = 231), Wang et al^11^ (*n* = 57), and Neftel et al^5^ (*n* = 9). The last 2 columns correspond to bulk RNA-seq datasets with DNA-derived purity labels: EORTC (*n* = 235) and TCGA (*n* = 144). Each panel illustrates the correlation between the predicted purity (y-axis) and ground truth or DNA-derived purity (x-axis). GBmap served as the training dataset for all tools except PUREE, which does not require reference data. Performance metrics, including correlation coefficients and error rates, are summarized in Table 2. Abbreviation: CNA, copy number alteration.

GBMPurity demonstrated strong performance across multiple validation datasets, depicted in Figure 2 and detailed in Table 2. Specifically, the model achieved an MAE of 0.15 on both the Wang and Neftel pseudobulk data, with a CCC of 0.88 and 0.77, respectively, demonstrating the high accuracy of our model. This extends to comparable correlations with DNA-based purity estimates of bulk tumors, with an MAE of 0.13 on both the TCGA and EORTC cohorts and CCCs of 0.60 and 0.74, respectively.

**Table 2.** Purity Estimation Benchmarking Results

Dataset	Model	MAE	RMSE	Pearson’s	CCC
GBmap*	*GBMPurityθ	**0.046**	**0.083**	**0.978**	**0.974**
	*MuSiCψ	0.105	0.163	0.937	0.921
	*CIBERSORTxψ	0.096	0.172	0.929	0.912
	PUREEλ	0.315	0.372	0.667	0.311
	*Scadenθ	0.378	0.403	0.010	0.001
Wang et al^11^	GBMPurityθ	**0.145**	**0.177**	**0.917**	**0.864**
	MuSiCψ	0.189	0.271	0.751	0.662
	CIBERSORTxψ	0.347	0.445	0.422	0.264
	PUREEλ	0.283	0.375	0.667	0.237
	Scadenθ	0.309	0.338	−0.082	−0.004
Neftel et al^5^	CIBERSORTxψ	**0.139**	**0.174**	0.815	**0.789**
	GBMPurityθ	0.162	0.186	**0.902**	0.687
	MuSiCψ	0.279	0.320	0.854	0.583
	PUREEλ	0.214	0.259	0.864	0.441
	Scadenθ	0.281	0.332	0.388	0.012
EORTC	GBMPurityθ	**0.128**	**0.160**	0.757	**0.743**
	MuSiCψ	0.138	0.181	0.725	0.709
	CIBERSORTxψ	0.170	0.229	0.581	0.570
	PUREEλ	0.167	0.212	**0.765**	0.529
	Scadenθ	0.195	0.229	−0.073	−0.005
TCGA	*PUREEλ	**0.102**	**0.123**	**0.803**	**0.701**
	MuSiCψ	0.114	0.160	0.730	0.694
	GBMPurityθ	0.133	0.159	0.690	0.597
	CIBERSORTxψ	0.160	0.200	0.677	0.565
	Scadenθ	0.272	0.299	−0.103	−0.003

Greek superscripts refer to methodology:

θ, deep learning based on pseudobulks;

ψ, machine learning weighting of single-cell reference profiles;

λ, pre-trained pan-cancer model based on genomic estimates. More details on these methods can be found in the *Methods* section. Asterisks refer to models trained on the respective dataset. Abbreviations: MAE, mean absolute error; RMSE, root mean squared error; CCC, correlation concordance coefficient; R Pearson’s correlation coefficient. Models are ordered within each dataset by descending CCC, with bold text indicating the best-performing metric for that dataset.

We then investigated the robustness of GBMPurity. We first evaluated the stability of the model by training GBMPurity with different weight initializations and observed consistency in model performance across the 3 single-cell datasets (Supplementary Figure S4A).

A tendency to underestimate purity was observed in the validation data (Supplementary Figure S4B). On further investigation, we found that the model underestimates purity linearly with increasing missing genes (Supplementary Figure S4C). Our validation data had 159 genes that were expressed beneath the limit used as a cutoff for feature selection (Supplementary Figure S4D), which accounts for the reduced purity estimations. As a result, we have added warnings to the web server for datasets that are missing >1% of genes, and the model won’t run with datasets missing 20% of required genes. Estimations for the Neftel data were resolved when using the integrated single-cell data and purity labels present in the GBmap resource (Supplementary Figure S4E).

### Benchmarking

We evaluated GBMPurity’s performance against several established purity estimation tools. PUREE^19^ was selected as a reference-free pan-cancer benchmark. CIBERSORTx^16^ and MuSiC^17^ were also chosen since these are established single-cell reference deconvolution tools. Finally, we incorporated an additional machine learning model, Scaden,^18^ trained on simulated pseudobulks, due to the methodological similarities with the training of GBMPurity. We opted not to include a wide range of deconvolution tools, as the selected models have already undergone extensive benchmarking in their respective studies. See Table 2 and *Methods* for descriptions of these tools.

Mean absolute error, RMSE, Pearson correlation, and CCC performance metrics were assessed across 5 datasets: 3 pseudobulk datasets with ground truth purity labels (GBmap, Wang et al, and Neftel et al) and 2 bulk RNA-seq datasets with DNA-derived purity estimates (TCGA and EORTC). The results are summarized in Figure 2 and Table 2. GBMPurity performed consistently well relative to other tools across all evaluation metrics in both validation modalities, demonstrating a particular efficacy compared to other methods at accurately identifying lower purity samples. Scaden exhibited notably poor performance, with all predictions deviating only slightly from 0.5. Despite rigorous investigations aimed at identifying potential issues in our preprocessing and training protocols, no discrepancies were found. This highlights the inherent challenges associated with the implementation of certain deconvolution methodologies.

### Model Interpretation

**Figure 3.**
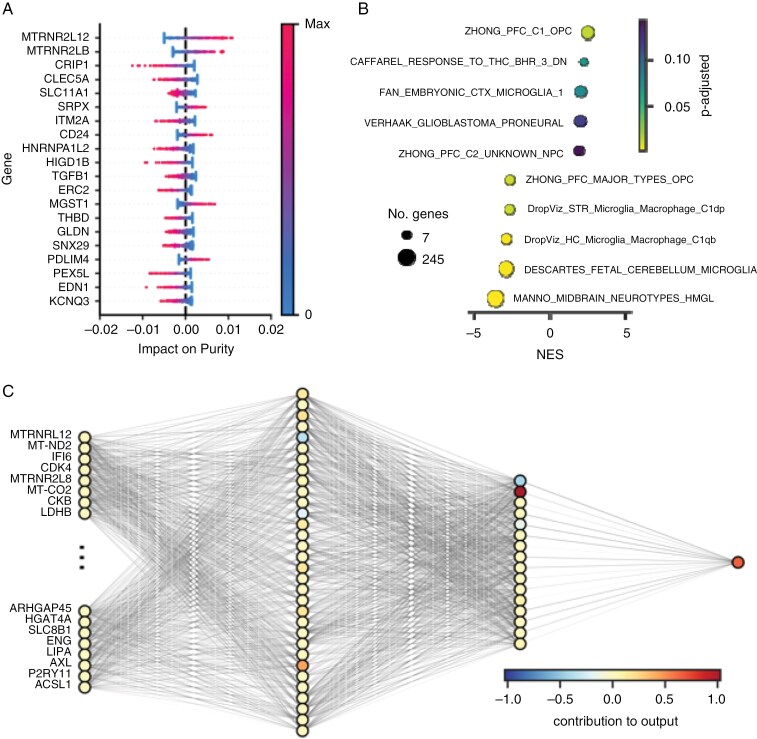
Interpretation and architecture analysis of GBMPurity. (A) SHapley Additive exPlanations (SHAP) summary plot illustrating the top 20 most influential features affecting model predictions in the pseudobulked GBmap data. The plot indicates the direction and magnitude of each feature’s impact on the predicted purity. (B) Gene set enrichment analysis (GSEA) of genes ranked by their average SHAP values across the pseudobulked GBmap data, utilizing a brain-specific gene set database (Hagenauer et al^39^). (C) Visualization of the GBMPurity model architecture, with neurons shaded based on their average contribution to purity estimation as determined by conductance analysis (Dhamdhere et al^41^).

Despite the inherent complexity of deep learning models, we sought to interpret GBMPurity to validate its predictions and derive biological insights. We applied SHAP^38^—which quantifies the importance of each input feature for a prediction—to the pseudobulks of the training GBmap dataset (Figure 3A). The use of dropout and weight decay to prevent overfitting resulted in a distribution of small impacts across many genes. MT-RNR2 like 12 (*MTRNR2L12*) and MT-RNR2 like 8 (*MTRNR2L8*) emerged as the most influential features, both contributing to higher purity estimates. As pseudogenes, the literature on these species is sparse, but their function is estimated to be involved in the negative regulation of apoptosis, a hallmark of cancer.^46^ Cysteine rich protein 1 (*CRIP1*), which is predominantly expressed in blood and immune cells,^47^ was the most influential gene associated with lower purity estimates.

We then ranked genes based on the average magnitude of their SHAP contributions over the pseudobulked training data, emphasizing broad average impact rather than rare, high-magnitude impacts. This ranking was used for GSEA with a curated database of brain-related gene sets.^39^ Gene set enrichment analysis identified that genes associated with neurodevelopmental precursors positively influenced purity estimates, while microglia and neuronal gene sets negatively impacted purity estimates (Figure 3B). These results instill confidence in our model as single-cell studies have shown that neoplastic GBM cells hijack neurodevelopmental processes,^48^ and microglia, as resident brain macrophages, along with neurons, the primary brain cell type, are key components of the nonneoplastic GBM microenvironment.

Conductance analysis was employed to quantify the importance of each node within the hidden layers.^41^ Visualization of GBMPurity and the importance of each node is shown in Figure 3C. This method can also be used to quantify the contribution of each input feature (ie, expression of associated genes) to specific nodes. Gene set enrichment analysis of these rankings suggested these nodes are polysemantic, not representing distinct identifiable biological modules (data not shown).

### Increased Normal Brain Infiltration in Proneural Tumors

**Figure 4.**
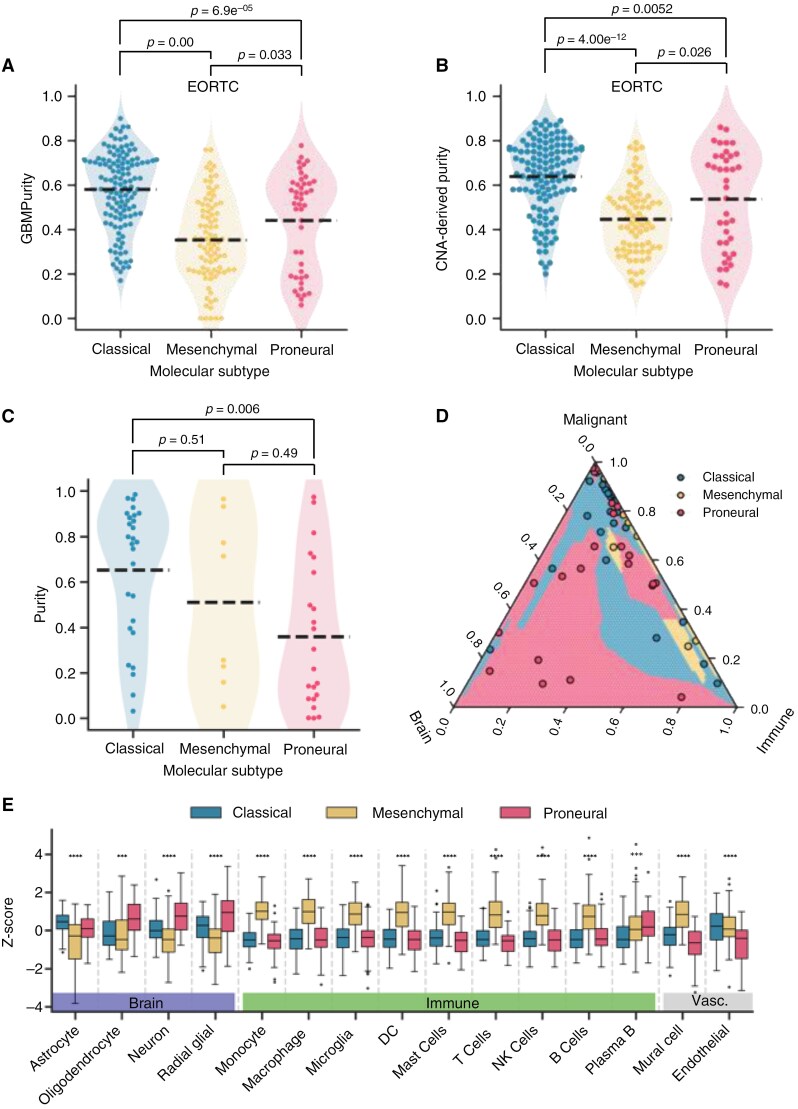
Proneural tumors exhibit a reduction in purity driven by increased normal brain cells. (A and B) Beeswarm plots showing (A) GBMPurity RNA-based purity estimates (*n* = 260) and (B) DNA-based purity estimates (*n* = 235) for the EORTC cohort bulk IDH^wt^ primary GBM tumors, stratified by GBM molecular subtype. Dashed lines indicate sample means for each subtype. (C) Beeswarm plot of purity estimates from single-nuclei pseudobulks (Wang et al^11^) stratified by molecular subtype (*n* = 57). Significance in panels (A, B, and D) was assessed using paired *t*-tests. (D) Ternary plot illustrating the composition of brain cells, malignant cells, and immune cells of each molecular subtype in the single-nuclei pseudobulks from Wang et al^11^. The plot area is shaded based on the nearest samples, calculated using Euclidean distance on centralized log-ratio transformed data. (E) Boxplot of GBMDeconvoluteR scores (Ajaib et al^37^) for the EORTC cohort, stratified by molecular subtype. Asterisks indicate the significance level of Bonferroni-adjusted cell-type-wise ANOVA tests (****P < .0001; ***P < .001). Full results of pairwise Tukey post-hoc tests are presented in Supplementary Table 2. Abbreviations: DC, dendritic cells; NK, natural killer.

To validate the inferences of GBMPurity against established biological knowledge, we inspected GBMPurity-derived purity estimates of the EORTC cohort across the molecular subtypes of GBM. Glioblastoma tumors can be stratified into classical, mesenchymal, and proneural molecular subtypes, each associated with distinct biological characteristics.^3,8^ Our model corroborated that mesenchymal tumors exhibit notably lower purity levels, which is consistent with previous findings^8^ (Figure 4A). However, we also observed a similar reduction in purity in the proneural subtype relative to the classical, which contrasts the aforementioned study. This finding was consistent within primary and recurrent tumors and irrespective of tumor location (Supplementary Figure S5A–C). Importantly, stratifying our validation pseudobulked single-nuclei data by molecular subtype demonstrates this finding isn’t due to a bias in GBMPurity underestimating the purity of proneural tumors relative to the other subtypes (Supplementary Figure S5D).

This finding was further validated by GBMPurity’s RNA-based purity estimates across 3 independent datasets: discovery, CGGA, and TCGA (Supplementary Figure S6A–C). DNA-based estimates from the EORTC cohort also supported this result (Figure 4B). However, the TCGA DNA-based purity estimates did not corroborate the finding, as seen in^8^ (Supplementary Figure S6D). We further investigated this finding by subtype-calling of snRNA-seq pseudobulk data. This also demonstrated a reduction in the purity of proneural tumors relative to the classical in the Wang et al data (Figure 4C), which was also validated in the GBmap snRNA-seq samples (Supplementary Figure S6E). However, it is worth noting that single-nuclei extraction itself has compositional biases, particularly underrepresenting immune cells.^49^

Interestingly, these proneural tumors had significantly increased compositions of nonmalignant brain cells (Figure 4D; Supplementary Figure S6F). We also investigated the source of reduced Proneural purity in our bulk samples. GBMDeconvoluteR,^37^ which provides cell-type-specific deconvolution scores for bulk RNA-seq data, revealed that proneural tumors have significantly increased scores for normal brain cell types, whereas mesenchymal tumors have a significant increase in immune cell types (Figure 4E; Supplementary Table S2).

## Discussion

In this study, we present GBMPurity, a novel deep learning tool tailored to estimate tumor purity from bulk RNA-seq data, specific to GBM. We trained a multilayer perceptron using the extensive scRNA-seq atlas, GBmap.^32^ This training data was enhanced by simulating pseudobulk samples, enabling the application of a more sophisticated model.

GBMPurity demonstrated robust performance in accurately predicting tumor purity across multiple validation datasets. The model achieved high CCC of 0.88 and 0.77 on the Wang and Neftel datasets, respectively, surpassing established deconvolution tools CIBERSORTx,^16^ MuSiC,^17^ PUREE,^19^ and Scaden.^18^ There was also good concordance to DNA-based purity estimates of matched bulk tumors.

GBMPurity offers 2 main advantages. First, it is a “plug-and-play” web-based tool that simplifies the process for GBM researchers, making sophisticated purity estimation accessible even to those with limited bioinformatics expertise. This tool can be applied to preexisting bulk RNA-seq datasets without requiring extensive computational resources or advanced technical knowledge. Second, the tailoring of this model to GBM has resulted in improved performance compared to general deconvolution and purity estimation methods.

Interpretation of this model also allows us to derive biological inferences. By employing SHAP for model interpretation, we identified key genes influencing tumor purity, providing insights that can guide further biological investigations. Notably, *MTRNR2L12* and *MTRNR2L8* emerged as the most influential genes in purity estimations. These are isoforms of the *MT-RNR2* gene, which encodes Humanin—a small peptide with neuroprotective and antiapoptotic activity in neuroblasts of Alzheimer’s diseased brains—and has recently been attributed with oncogenic effects in glioblastoma cells ^50–52^.

GBMPurity’s inference on bulk RNA-seq identified a significantly lower purity in mesenchymal and proneural subtypes relative to classical. While the association of the mesenchymal subtype with increased immune infiltrate is well documented,^9,53^ the lower purity in the proneural subtype relative to that of the classical has not yet been described. This finding was consistent across 5 bulk RNA-seq datasets and 2 snRNA-seq pseudobulk datasets.

Discrepancies between DNA- and RNA-derived purity estimates for proneural tumors in the TCGA cohort likely stem from DNA and RNA being extracted from distinct tumor slices. Given GBM’s intratumoral heterogeneity, slice-to-slice variations in purity are expected.^54^ Additionally, spatially distinct regions within a single lesion can represent different molecular subtypes. Since molecular subtypes are determined using RNA data, assigning an RNA-derived subtype to a DNA slice may result in misclassification if they represent different tumor regions.^55^ In contrast, no such discrepancy was observed in the EORTC cohort, where DNA and RNA were extracted from the same tumor slice and showed consistent agreement with reduced proneural purity compared to classical. However, differences in sequencing and purity estimation methods may contribute: EORTC used targeted sequencing of 287 cancer genes with solely CNA-based purity estimation, whereas TCGA employed whole-exome sequencing and the ABSOLUTE algorithm, which additionally incorporates mutation data.^27,56^

Deconvolution of bulk tumors suggests the reduced purity in the proneural subtype compared to classical can be attributed to the increased presence of normal resident brain cells, as opposed to the increased immune infiltrate of mesenchymal tumors. This was also validated in snRNA-seq data. The enriched association of, and interaction between, neoplastic GBM cells and normal brain cells within proneural tumors is now well documented^6,57,58^ alongside the repeated observation of mesenchymal cells being associated with infiltrating and brain-resident immune cells.^5,26^ Our results corroborate these findings, adding confidence to the accuracy of GBMPurity, and raising interesting insights that can be further elucidated by the research community. This underscores the potential of this tool to enhance the interpretation of bulk RNA-seq data and provide more accurate biological insights into GBM.

Applications of GBMPurity hold promise for advancing glioblastoma research by streamlining the analysis of bulk GBM omics data and offering a more pragmatic interpretation in the context of TME composition. This utility may also extend beyond the lab and into the clinic. For example, immunotherapies are a promising approach in GBM treatment, but as of yet, have produced variable responses.^59,60^ Applying GBMPurity to differentiate between responders and nonresponders to immunotherapy could enhance prognostic assessments, given the associations between TME composition and therapeutic response in other solid tumors.^61,62^

Future iterations of this model could facilitate automatic correction of the input matrix for estimated purity, enabling more targeted and consistent analyses of the malignant components in bulk omics data. Additionally, while our current approach selected genes from scRNA-seq data that are representative of our bulk RNA-seq data, incorporating batch correction methods—such as those implemented by CIBERSORTx^16^—could further enhance intermodal applicability. Moreover, varying the ratios of different cell types in the nonmalignant component of simulated pseudobulks could increase the diversity of the training data. The accuracy of GBMPurity may be improved further by utilizing state-of-the-art deep learning architectures such as transformers.^63^ Finally, our methodology is only restricted to GBM in the use of single-cell GBM RNA-seq data for training and therefore has the potential to be extended to other cancer types, offering a route toward a comprehensive pan-cancer purity estimation tool.

## Supplementary Material

noaf026_suppl_Supplementary_Figures

noaf026_suppl_Supplementary_Tables
