# Supplementary material for: GBMPurity: A machine learning tool for estimating glioblastoma tumor purity from bulk RNA-sequencing data
Source: Neuro Oncol. 2025 Feb 1;27(6):1458–73. doi: 10.1093/neuonc/noaf026 (PMC12309721; doi:10.1093/neuonc/noaf026)
Supplement: noaf026_suppl_Supplementary_Figures [file noaf026_suppl_supplementary_figures.pptx]

## Slide 1
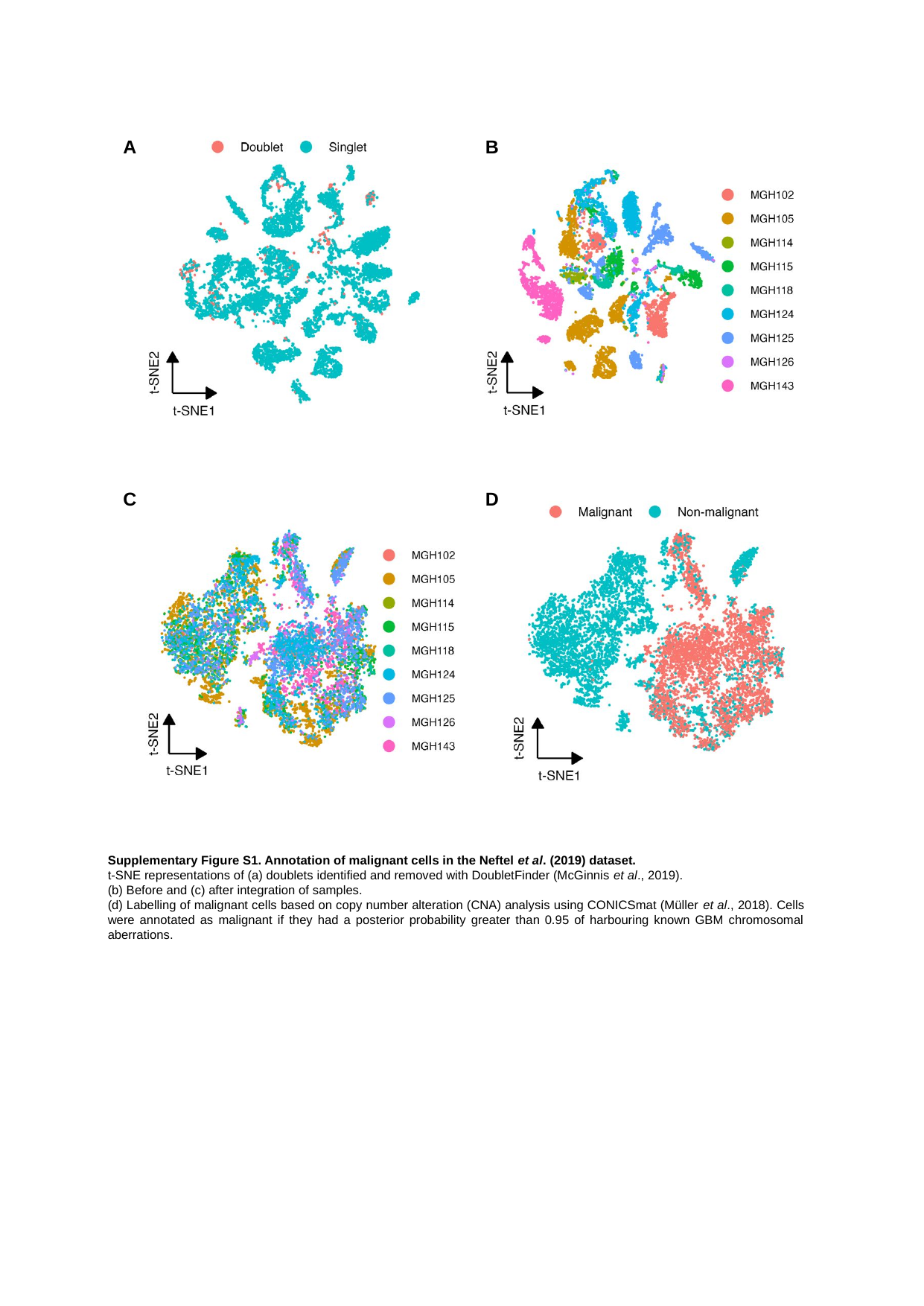

A
B
C
D
Supplementary Figure S1. Annotation of malignant cells in the Neftel et al. (2019) dataset.
t-SNE representations of (a) doublets identified and removed with DoubletFinder (McGinnis et al., 2019).
(b) Before and (c) after integration of samples.
(d) Labelling of malignant cells based on copy number alteration (CNA) analysis using CONICSmat (Müller et al., 2018). Cells were annotated as malignant if they had a posterior probability greater than 0.95 of harbouring known GBM chromosomal aberrations.

## Slide 2
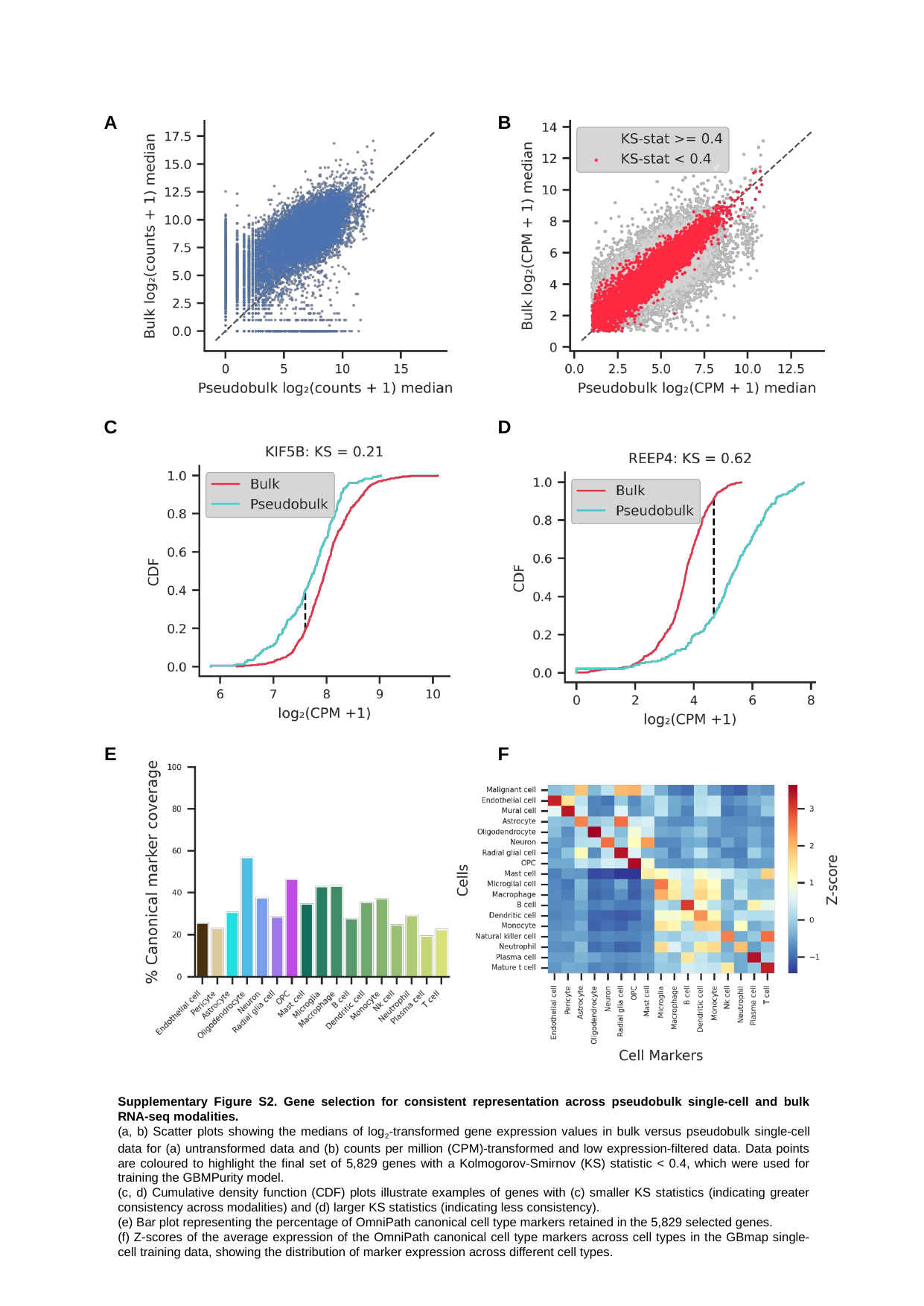

A
B
C
D
E
F
Supplementary Figure S2. Gene selection for consistent representation across pseudobulk single-cell and bulk RNA-seq modalities.
(a, b) Scatter plots showing the medians of log2-transformed gene expression values in bulk versus pseudobulk single-cell data for (a) untransformed data and (b) counts per million (CPM)-transformed and low expression-filtered data. Data points are coloured to highlight the final set of 5,829 genes with a Kolmogorov-Smirnov (KS) statistic < 0.4, which were used for training the GBMPurity model.
(c, d) Cumulative density function (CDF) plots illustrate examples of genes with (c) smaller KS statistics (indicating greater consistency across modalities) and (d) larger KS statistics (indicating less consistency).
(e) Bar plot representing the percentage of OmniPath canonical cell type markers retained in the 5,829 selected genes.
(f) Z-scores of the average expression of the OmniPath canonical cell type markers across cell types in the GBmap single-cell training data, showing the distribution of marker expression across different cell types.

## Slide 3
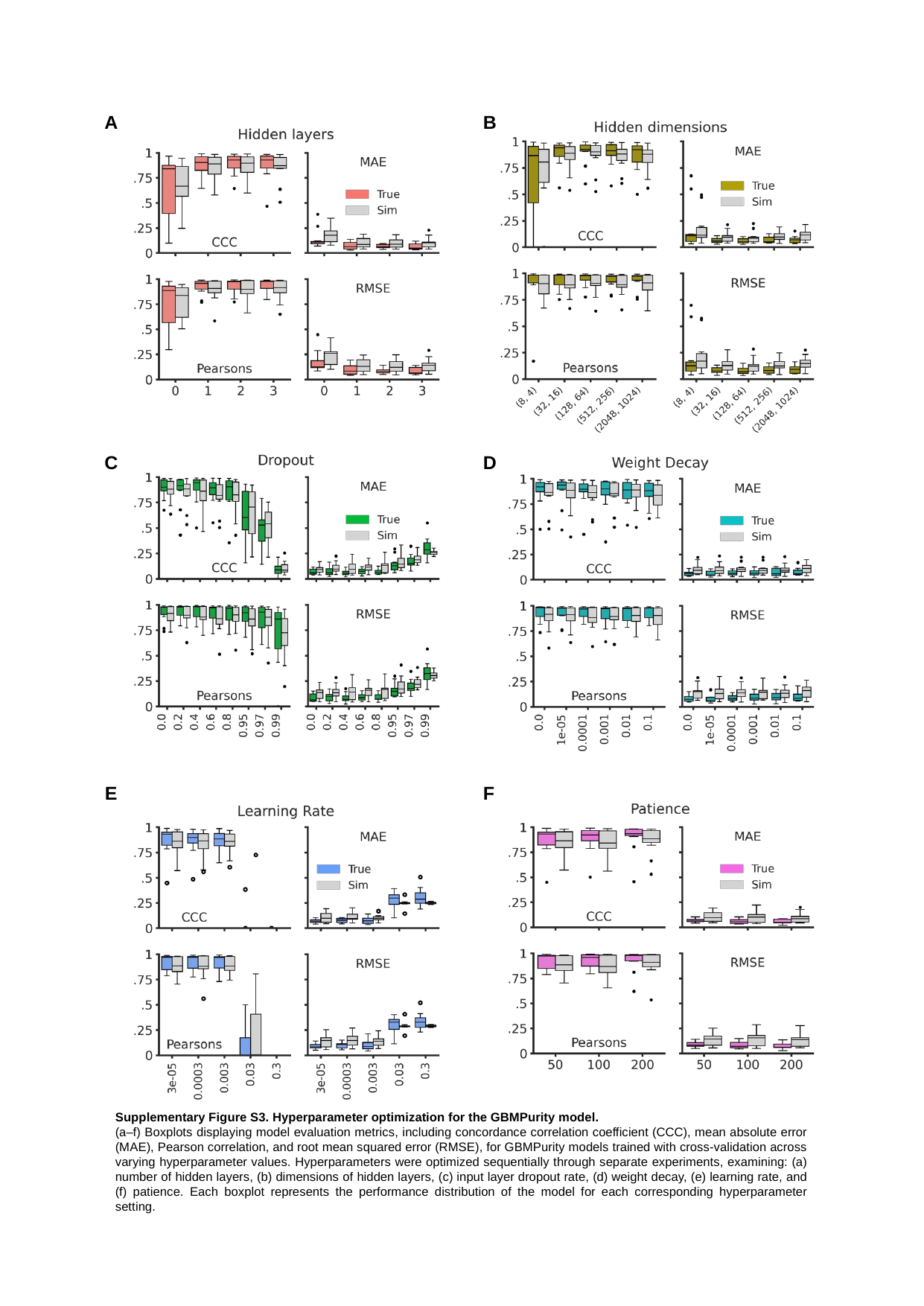

A
B
C
D
E
F
Supplementary Figure S3. Hyperparameter optimization for the GBMPurity model.
(a–f) Boxplots displaying model evaluation metrics, including concordance correlation coefficient (CCC), mean absolute error (MAE), Pearson correlation, and root mean squared error (RMSE), for GBMPurity models trained with cross-validation across varying hyperparameter values. Hyperparameters were optimized sequentially through separate experiments, examining: (a) number of hidden layers, (b) dimensions of hidden layers, (c) input layer dropout rate, (d) weight decay, (e) learning rate, and (f) patience. Each boxplot represents the performance distribution of the model for each corresponding hyperparameter setting.

## Slide 4
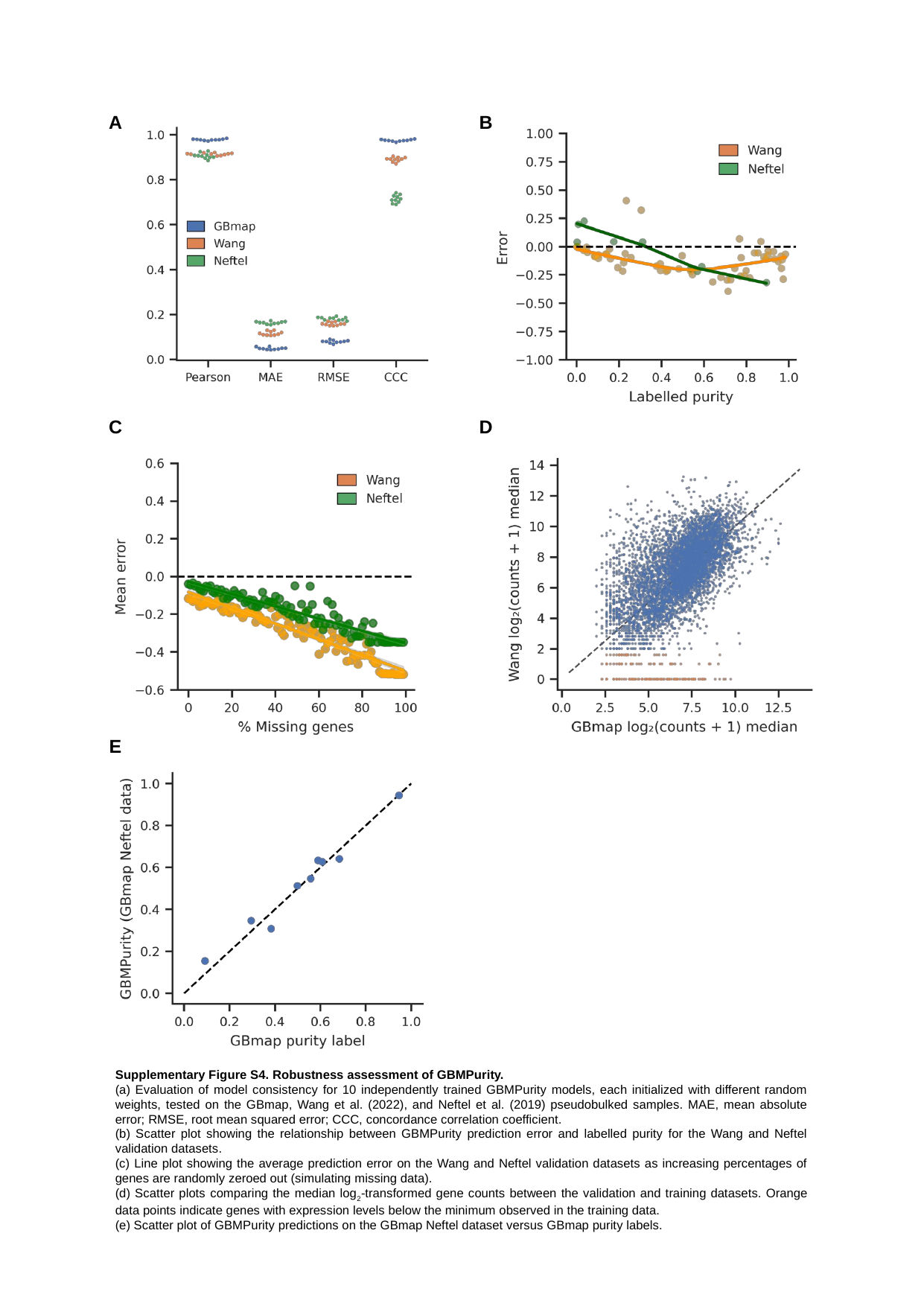

A
B
C
D
E
Supplementary Figure S4. Robustness assessment of GBMPurity.
(a) Evaluation of model consistency for 10 independently trained GBMPurity models, each initialized with different random weights, tested on the GBmap, Wang et al. (2022), and Neftel et al. (2019) pseudobulked samples. MAE, mean absolute error; RMSE, root mean squared error; CCC, concordance correlation coefficient.
(b) Scatter plot showing the relationship between GBMPurity prediction error and labelled purity for the Wang and Neftel validation datasets.
(c) Line plot showing the average prediction error on the Wang and Neftel validation datasets as increasing percentages of genes are randomly zeroed out (simulating missing data).
(d) Scatter plots comparing the median log2-transformed gene counts between the validation and training datasets. Orange data points indicate genes with expression levels below the minimum observed in the training data.
(e) Scatter plot of GBMPurity predictions on the GBmap Neftel dataset versus GBmap purity labels.

## Slide 5
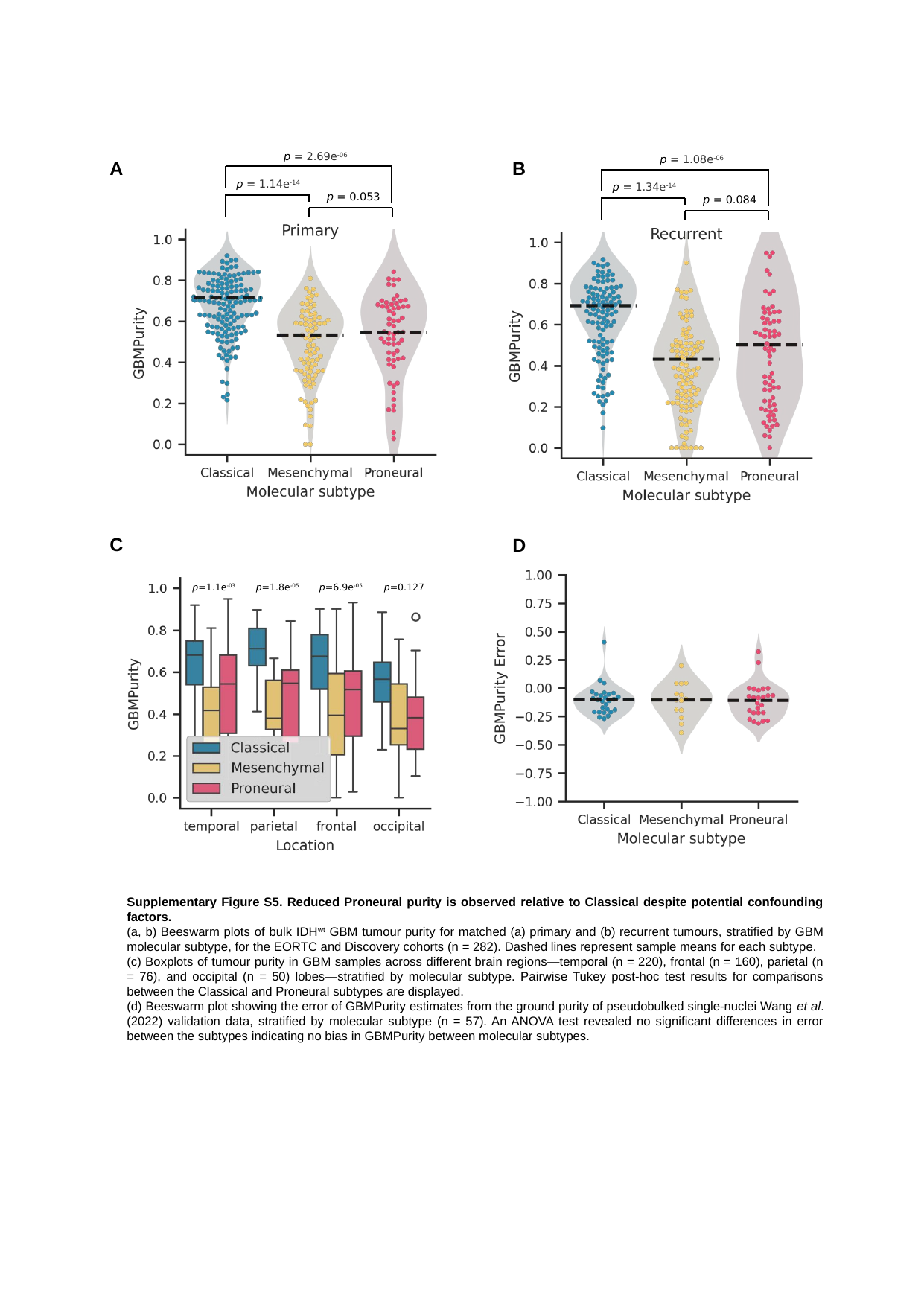

p = 2.69e-06
p = 1.14e-14
p = 0.053
p = 1.08e-06
p = 1.34e-14
p = 0.084
A
B
C
D
p=1.1e-03
p=1.8e-05
p=6.9e-05
p=0.127
Supplementary Figure S5. Reduced Proneural purity is observed relative to Classical despite potential confounding factors.
(a, b) Beeswarm plots of bulk IDHwt GBM tumour purity for matched (a) primary and (b) recurrent tumours, stratified by GBM molecular subtype, for the EORTC and Discovery cohorts (n = 282). Dashed lines represent sample means for each subtype.
(c) Boxplots of tumour purity in GBM samples across different brain regions—temporal (n = 220), frontal (n = 160), parietal (n = 76), and occipital (n = 50) lobes—stratified by molecular subtype. Pairwise Tukey post-hoc test results for comparisons between the Classical and Proneural subtypes are displayed.
(d) Beeswarm plot showing the error of GBMPurity estimates from the ground purity of pseudobulked single-nuclei Wang et al. (2022) validation data, stratified by molecular subtype (n = 57). An ANOVA test revealed no significant differences in error between the subtypes indicating no bias in GBMPurity between molecular subtypes.

## Slide 6
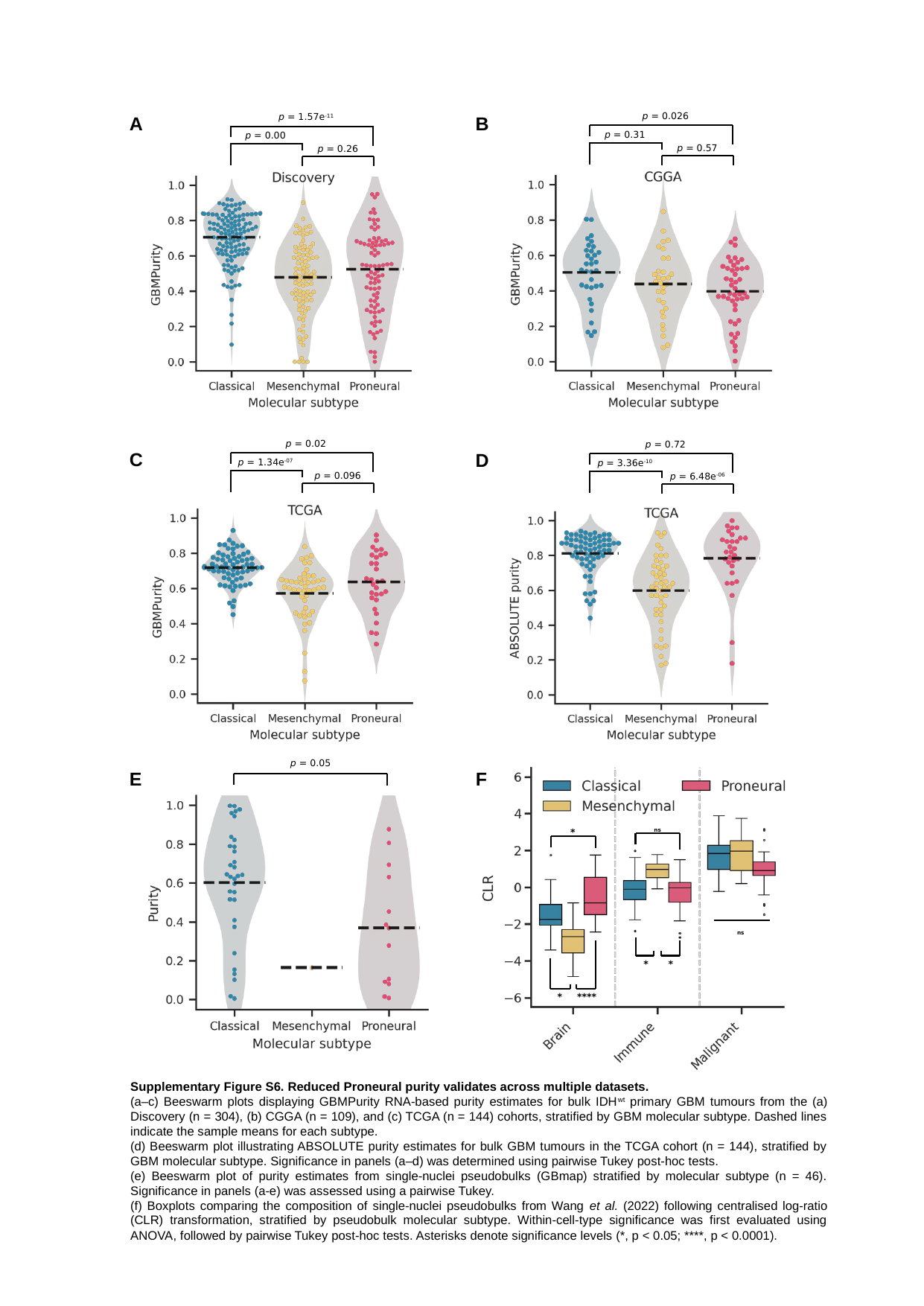

p = 0.026
p = 0.31
p = 0.57
p = 1.57e-11
p = 0.00
p = 0.26
A
B
p = 0.02
p = 1.34e-07
p = 0.096
p = 0.72
p = 3.36e-10
p = 6.48e-06
C
D
p = 0.05
E
F
ns
*
*
****
ns
*
*
Supplementary Figure S6. Reduced Proneural purity validates across multiple datasets.
(a–c) Beeswarm plots displaying GBMPurity RNA-based purity estimates for bulk IDHwt primary GBM tumours from the (a) Discovery (n = 304), (b) CGGA (n = 109), and (c) TCGA (n = 144) cohorts, stratified by GBM molecular subtype. Dashed lines indicate the sample means for each subtype.
(d) Beeswarm plot illustrating ABSOLUTE purity estimates for bulk GBM tumours in the TCGA cohort (n = 144), stratified by GBM molecular subtype. Significance in panels (a–d) was determined using pairwise Tukey post-hoc tests.
(e) Beeswarm plot of purity estimates from single-nuclei pseudobulks (GBmap) stratified by molecular subtype (n = 46). Significance in panels (a-e) was assessed using a pairwise Tukey.
(f) Boxplots comparing the composition of single-nuclei pseudobulks from Wang et al. (2022) following centralised log-ratio (CLR) transformation, stratified by pseudobulk molecular subtype. Within-cell-type significance was first evaluated using ANOVA, followed by pairwise Tukey post-hoc tests. Asterisks denote significance levels (*, p < 0.05; ****, p < 0.0001).
